# The CNGRC-GG-_D_(KLAKLAK)_2_ peptide induces a caspase-independent, Ca^2+^-dependent death in human leukemic myeloid cells by targeting surface aminopeptidase N/CD13

**DOI:** 10.18632/oncotarget.6523

**Published:** 2015-12-09

**Authors:** Sandrine Bouchet, Ruoping Tang, Fanny Fava, Ollivier Legrand, Brigitte Bauvois

**Affiliations:** ^1^ Centre de Recherche des Cordeliers, INSERM UMRS1138, Sorbonne Universités UPMC Paris 06, Université Paris Descartes Sorbonne Paris Cité, Paris, France; ^2^ Assistance Publique des Hôpitaux de Paris, Paris, France; ^3^ Centre de Recherche de Saint-Antoine, INSERM UMRS 938, Service d'Hématologie, Hôpital St Antoine, Paris, France; ^4^ Sorbonne Universités UPMC Paris 06, Paris, France

**Keywords:** calcium, leukemia, metalloproteinase, necrosis, superoxide radical

## Abstract

The CD13 antigen's binding site for the Asn-Gly-Arg (NGR) motif enables NGR-containing chemotherapeutic drugs to be delivered to CD13-positive tumours. Human CD13-positive acute myeloid leukemia (AML) cells proliferate abnormally and escape death. Here, we show that the CNGRC-GG-_D_(KLAKLAK)_2_ peptide induces death in AML cell lines (U937, THP-1, NB4, HL-60) and primary blood cells from AML patients. Cell death was characterized as a caspase-independent mechanism, without DNA fragmentation, but phosphatidylserine externalization and membrane disruption. Our results demonstrate in U937 cells that (i) the NGR-peptide triggers the loss of mitochondrial potential(ΔΨm) and generates superoxide anion (O_2_^−^), (ii) N-acetyl-L-cysteine (NAC) and extra/intracellular Ca^2+^ chelators (BAPTA) prevent both O_2_− production and cell death, (iii) the Ca^2+^-channel blocker nifedipine prevents cell death (indicating that Ca^2+^ influx is the initial death trigger), and (iv) BAPTA, but not NAC, prevents ΔΨm loss (suggesting O_2_^−^ is a mitochondrial downstream effector). AML cell lines and primary blasts responding to the lethal action of NGR-peptide express promatrix metalloproteinase-12 (proMMP-12) and its substrate progranulin (an 88 kDa cell survival factor). A cell-free assay highlighted proMMP-12 activation by O_2_^−^. Accordingly, NGR-peptide's downregulation of 88 kDa progranulin protein was prevented by BAPTA and NAC. Conversely, AML blast resistance to NGR-peptide is associated with the expression of a distinct, 105 kDa progranulin isoform. These results indicate that CNGRC-GG-_D_(KLAKLAK)_2_ induces death in AML cells through the Ca^2+^-mitochondria-O_2_.-pathway, and support the link between proMMP-12 activation and progranulin cleavage during cell death. Our findings may have implications for the understanding of tumour biology and treatment.

## INTRODUCTION

The antigen CD13/membrane-anchored amino-peptidase-N (APN) (EC 3.4.11.2) is expressed on the surface of epithelial cells, fibroblasts and myeloid cells [1, 2]. CD13 is dysregulated in several solid and haematological tumours and is thus considered to be a useful biomarker [1, 2]. Moreover, CD13 is abnormally expressed on the endothelial cells of the angiogenic vasculature but not those of the normal vasculature [1]. It was initially demonstrated that the Asn-Gly-Arg (NGR) motif binds to CD13-positive blood vessels in tumours but not to epithelia in the normal kidney or other CD13-rich tissues [3]. This selectivity might be related to different CD13 isoforms (with differential glycosylation or conformations) [3]. Therefore, NGR-targeted drugs might enhance drug delivery to various solid tumours and tumour-associated angiogenic blood vessels [3–8]. A large variety of molecules have been coupled to the NGR motif (which can be flanked by two cysteine moieties in a circular CNGRC peptide), including cytotoxic agents (doxorubicin, 5′ fluoro-2′-deoxyuridine, 5-fluorouracil, pingyangmycin), human cytokines (TNF-α and IFN-γ) and anti-angiogenic drugs (such as endostatin and _D_(KLAKLAK)_2_) [2, 3, 7, 9–12]. The CNGRCG motif binds to the APN enzymatic active site but it resists APN degradation [13]. Most studies in animal models indicate that NGR-linked drugs exhibit tumour-homing properties and anticancer activity [3, 9] In mice and rabbits, the immunogenicity of the NGR motif (whether alone or conjugated to a drug) appears to be very low [3]. CNGRC-TNF-α has already been tested (both as a single agent and in combination with chemotherapy) in Phase I, II and III clinical trials in patients with various solid tumours [14, 15]. The trials' results indicate stabilization in 50% of the patients treated. Weekly dosing maintained this stabilisation for a median time of more than 9 months, with limited toxicity - thus suggesting that a peptide-based tumour targeting approach is viable [14, 15]. The CNGRCG-TNF-α compound fails to bind to CD13 expressed on human myeloid cells (e.g. the THP-1 cell line and blood monocytes), suggesting that the NGR-targeted drug approach might not be valid in myeloid cells [16]. However, it has not been established whether other NGR-ligands (such as NGR- _D_(KLAKLAK)_2_) can affect myeloid cells in general and acute myeloid leukemia cells in particular.

Acute myeloid leukemia (AML) is a clinically and genetically heterogeneous hematopoietic cancer characterized by the clonal accumulation of immature myeloid precursors in the bone marrow [17]. Human AML cells show abnormally high levels of proliferation and survival, and infiltrate extramedullary organs [17]. The conventional chemotherapeutic approach to treatment of AML patients is based on combining an anthracycline with cytarabine [18]. Although the majority of AML cases respond to initial treatment, relapse is frequent and emphasizes the malignant cells' resistance to chemotherapy [17]. The CD13 antigen is strongly expressed on stem cells and leukemic blasts in all AML subtypes [19]. We previously showed that anti-CD13 monoclonal antibodies (mAbs) have the ability to induce apoptosis in AML cells, related to the intertwined activation of PI3K and AKT kinases involved in signal transduction and caspases involved in the intrinsic and extrinsic pathways of apoptosis [20]. Hence, CD13 may be a pro-apoptotic target in this disease. Considering the risk that mAbs may induce a mechanism-dependent toxicity that can add to therapeutic activity as exemplified by the use of gemtuzumab ozogamicin in AML [21], we therefore investigated the possibility to induce the death of AML cells with the CNGRC-GG-_D_(KLAKLAK)_2_ peptide by targeting leukemic CD13. _D_(KLAKLAK)_2_ is a cationic a-helix peptide originally designed as an antibacterial peptide [22]. Antibacterial peptides selectively kill bacteria while maintaining low mammalian cell cytotoxicity. Such selectivity has been attributed to plasma membrane differences between bacteria and mammalian cells, those of bacteria being negatively charged whereas mammalian membranes are generally neutral [23]. Indeed, _D_(KLAKLAK)_2_ shows no toxic effects on various human endothelial, epithelial and hematopoietic cell lines [24, 25]. Using a rat liver mitochondria-dependent cell free system, Ellerby et al. have demonstrated that _D_(KLAKLAK)_2_ induces mitochondrial swelling and caspase-3 activation [9]. The mechanism is based on the electrostatic binding of peptide's cationic amino-acids to mitochondria's anionic phospholipids, resulting in the loss of mitochondrial membrane potential [9]. Apart the NGR motif recognizing CD13 [9], several other cancer-cell binding peptides have been conjugated to _D_(KLAKLAK)_2_, including the protein transduction domain (PTD-5) targeting head and neck tumors [26, 27], the cell internalizing motif F^F^/_Y_XLRS targeting neuropilin-1 leukemia and lymphoma cells [24], the cell binding peptide LTVSPWY and the gastrin-releasing peptide GNHWAVGHLM targeting breast cancer cells [28], the cancer recognition peptide LTVSPWY targeting a variety of tumor epithelial cell lines [29], and the synthetic Toll-like receptor 2-mediated cell-penetrating peptide targeting AML cells [25]. Most resulting fusion peptides induce cell death as evidenced by plasma membrane blebbing, phosphatidylserine externalization and/or procaspase-3 activation [9, 25–28]. In addition, the fusion peptide consisting of PTD-5 and _D_(KLAKLAK)_2_ induces the release of cytochrome c from mitochondria accompanied by production of H_2_O_2_ [27]. In this study, we investigated the effects of the CNGRC-GG-_D_(KLAKLAK)_2_ peptide on AML cell lines *in vitro* and AML patients' cells *ex vivo*. Our results indicate that this peptide induces death in AML cells in a NGR-dependent manner. The underlying mechanisms and intracellular signaling pathways triggered by this peptide in AML cells were investigated.

## RESULTS

### Induction of cell death in AML cell lines by CNGRC-GG-_D_(KLAKLAK)_2_

**Figure 1:**
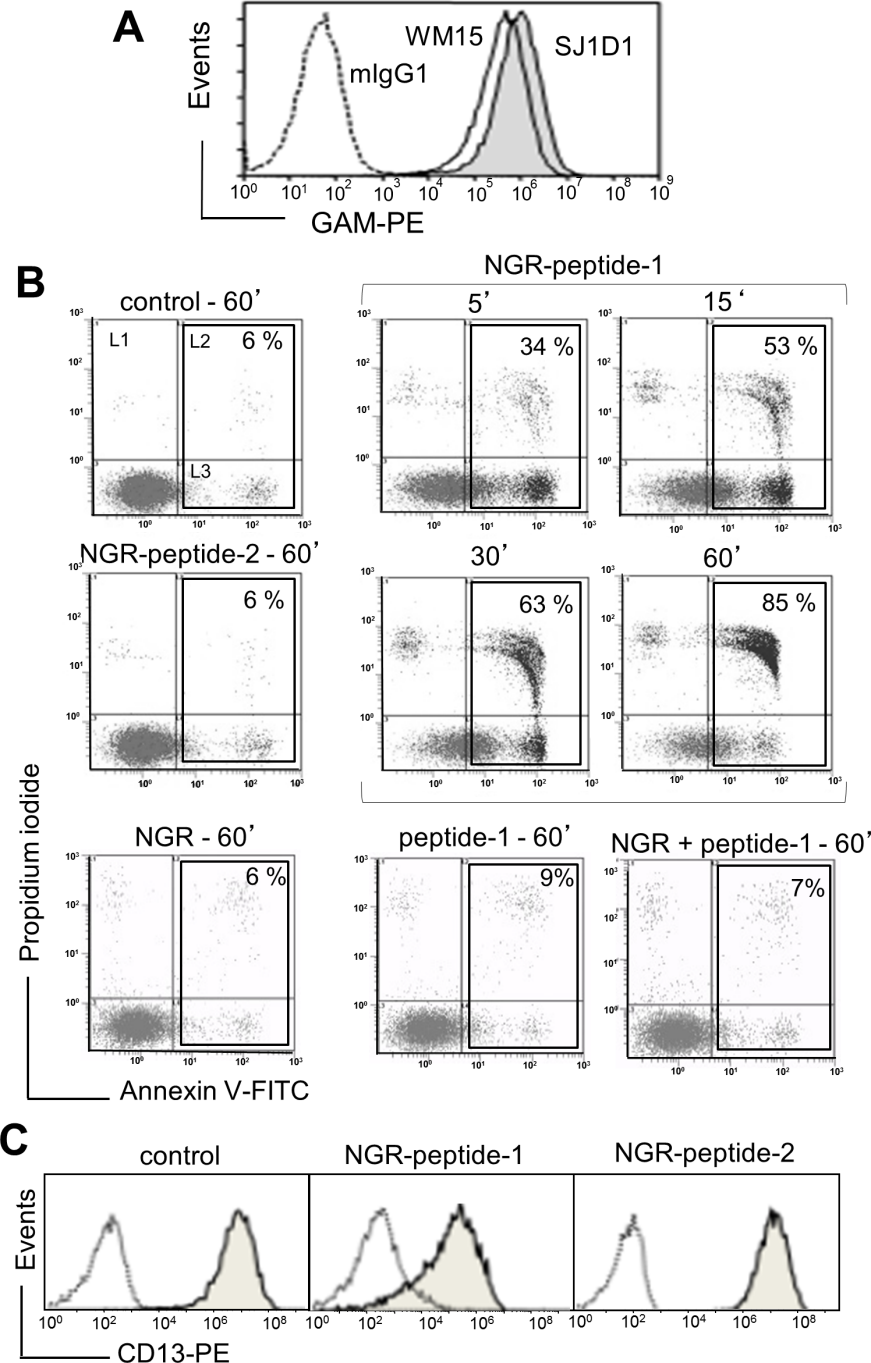
The CNGRC-GG-D(KLAKLAK)_2_ peptide induces cell death in U937 cells (**A**) U937 cells were incubated with anti-CD13 mAbs (SJ1D1 or WM15) or mIgG1 (negative control), stained with GAM-PE and then analyzed for surface CD13 expression by flow cytometry. (**B**) U937 cells (2 × 10^5^/ml) were treated with 50 μM CNGRC-GG-_D_(KLAKLAK)_2_ (NGR-peptide-1) or 50 μM CNGRCGG-LVTT-OH (NGR-peptide-2, the negative control) or 50 μM CNGRC-G (NGR) or 50 μM _D_(KLAKLAK)_2_ (peptide-1) or a combination of 25 μM NGR and 25 μM peptide-1for the indicated times. Detection of cell death after annexin-V-FITC/PI staining and flow cytometry. Results are expressed as the log PI fluorescence intensity (on the y axis) vs. log annexin-V-FITC fluorescence intensity (on the x axis). Dead cells are highlighted in the box and the proportion (L2 + L3 gates) is shown in the Figure. One representative experiment is shown here, and experiments were repeated at least four times. (**C**) U937 cells (2 × 10^5^/ml) were cultured for 10 min in the presence of absence of NGR-peptides (50 μM). Cells were stained with mIgG1-PE (negative control; white peak) or anti-CD13 (SJ1D1)-PE (grey peak) and then examined by flow cytometry.

**Figure 2:**
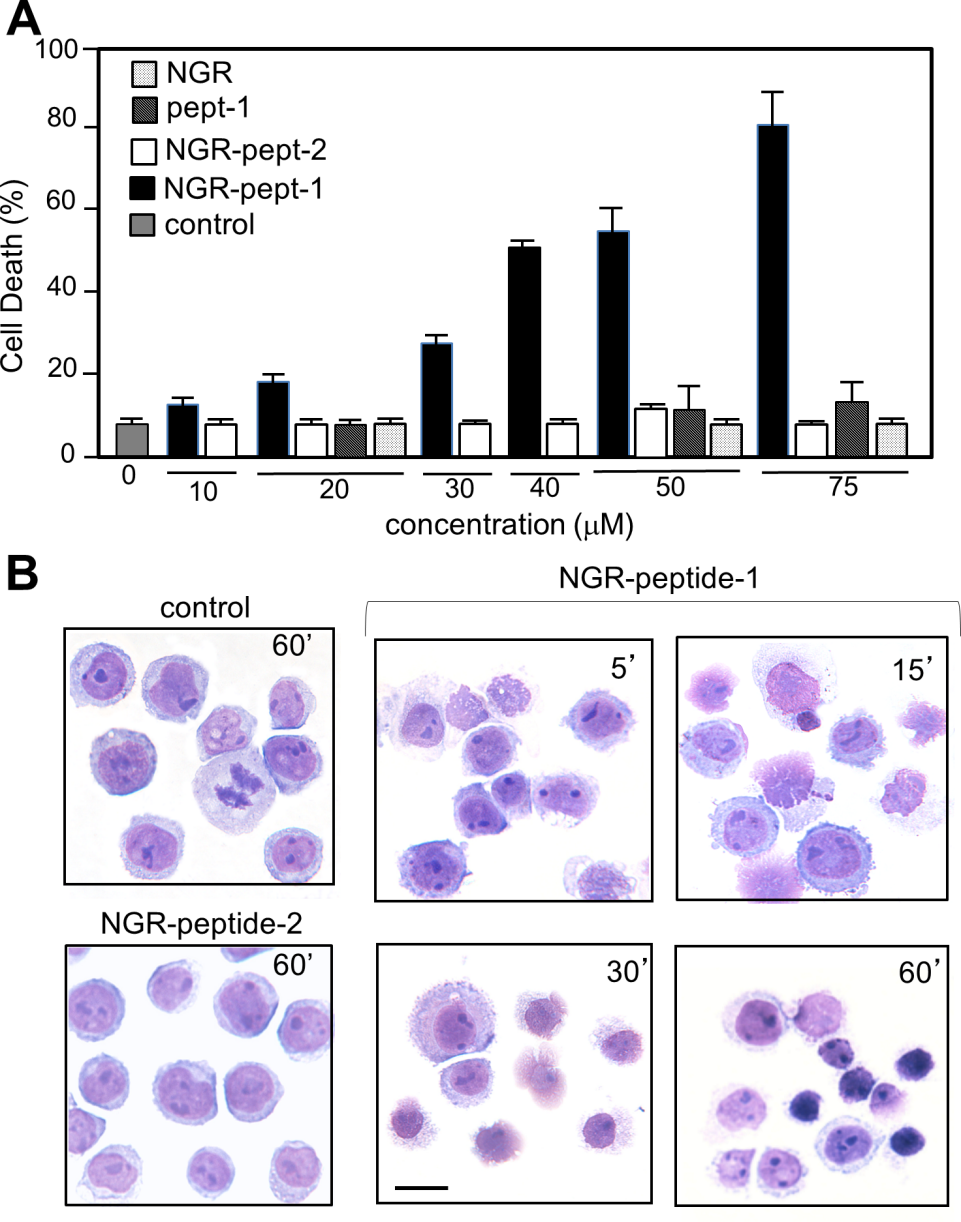
The CNGRC-GG-_D_(KLAKLAK)_2_ peptide induces cell death in U937 cells U937 cells (2 × 10^5^/ml) were treated with CNGRC-GG-_D_(KLAKLAK)_2_ (NGR-peptide-1), CNGRCGG-LVTT-OH (NGR-peptide-2, the negative control), NGR or free peptide-1 for 15 min and at the indicated concentrations. Detection of cell death (L2 + L3 gates) as detailed in Figure 1. The values correspond to the mean ± SD of four independent experiments. (**B**) Light microscopy of non-treated, NGR-peptide-1- and NGR-peptide-2-treated U937 cells. Original magnification: x 600. May Grünwald stain. Scale bar: 10 μm.

**Figure 3:**
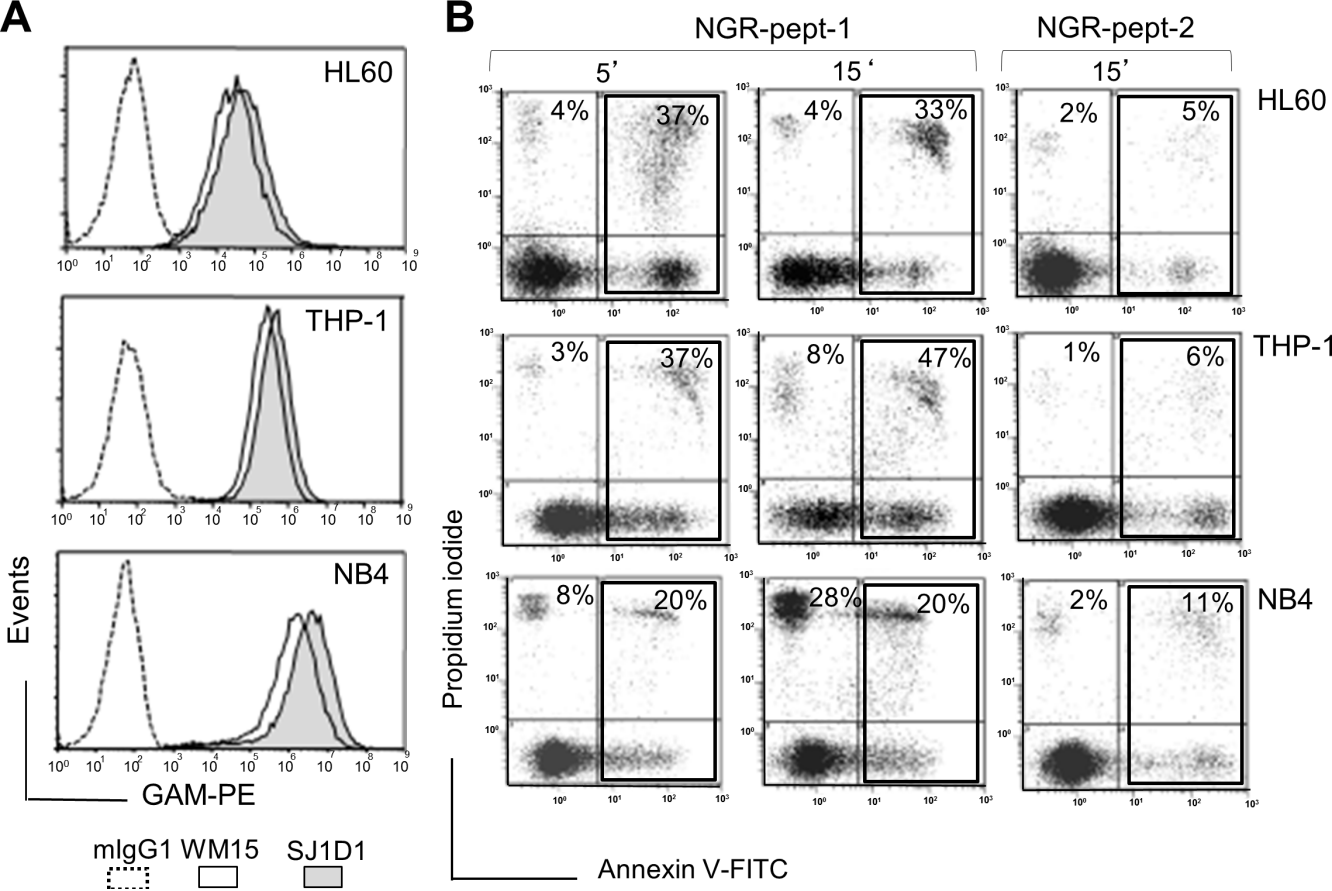
Expression of CD13 on AML cell lines and their sensitivity to NGR-peptide-1 (**A**) HL-60, THP-1 and NB4 cells were stained with anti-CD13 mAbs (SJ1D1 or WM15) as detailed in Figure 1A. (**B**) Cells lines (2 × 10^5^/ml) were treated with 50 μM NGR-peptides for 5 and 15 min. Detection of dead cells after annexin-V-FITC/PI staining and flow cytometry. Annexin-V-positive and PI-positive cells are highlighted in the box and their percentage is shown in the Figure.

As previously shown [20], the specific mAbs against CD13, WM15 (obtained after immunization with blasts from one AML patient [30]) and SJ1D1 (raised against CD13 from the myeloid cell line KG1 [31]) labelled to a similar extent CD13 expressed on the surface of AML (monoblastic, M5) U937 cells (Figure 1A). WM15 neutralizes CD13′s enzyme activity [30] and sterically overlaps (at least in part) the NGR binding site on human CD13 [32]. We first examined the effects of CNGRC-GG-_D_(KLAKLAK)_2_ (hereafter referred to as NGR-peptide-1), CNGRC-GG-LVTT (NGR-peptide-2, a negative control), CNGRCG (hereafter referred to as NGR) and _D_(KLAKLAK)_2_ (peptide-1) on the viability of U937 cells. Cell death was assessed by determining phosphatidylserine (PS) exposure at the cell surface (using annexin-V-FITC binding) and cell membrane disruption (using propidium iodide/PI labelling). Time-course studies revealed a rapid, time-dependent, lethal effect of NGR-peptide-1 on U937 cells, when compared with NGR-peptide-2 or the absence of treatment (Figure 1B). After 60 min, the majority of NGR-peptide-1-treated cells were stained by both annexin-FITC and PI - reflecting loss of the plasma membrane (Figure 1B). In contrast, as previously shown [24, 25], peptide-1 and NGR, alone or combined, show no toxic effects on U937 cells (Figure 1B). The lack of effects of NGR, peptide-1 and NGR-peptide-2 on U937 cell viability indicates that cell death occurs only when the NGR-motif is fused to peptide-1. In parallel, surface levels of CD13 in U937 cells fell (5 min treatment) on NGR-peptide-1-treated cells but did not change on non-treated and NGR-peptide-2-treated cells (Figure 1C). Decrease in CD13 surface expression likely reflected internalization of CD13. Cytochalasin D (inhibitor of actin filament polymerisation) and nocodazole (inhibitor of microtubules formation) did not influence NGR-peptide-1-induced U937 cell death and NGR-peptide-1-mediated CD13 downregulation, suggesting that CD13 was not internalized by phagocytosis. NGR-peptide-1's lethal effects were dose-dependent over the range 1–75 μM (Figure 2A). Accordingly, light microscopy observations revealed that NGR-peptide-1 treatment rapidly deteriorated the cell morphology in a manner indicative of cell death (Figure 2B). The numbers of shrunken cells with a damaged plasma membrane but an intact nucleus increased over time (Figure 2B). The other AML cell lines HL-60 (myeloblastic, M2), NB4 (promyelocytic, M3) and THP- 1 (monoblastic, M5) expressed surface CD13 (Figure 3A) and were also sensitive to the NGR-peptide-1's lethal effects as evidenced by cell shrinkage (data not shown), phosphatidylserine externalization and cell membrane disruption (Figure 3B).

### Induction of *ex vivo* death of AML patients' cells by NGR-peptide-1

**Figure 4:**
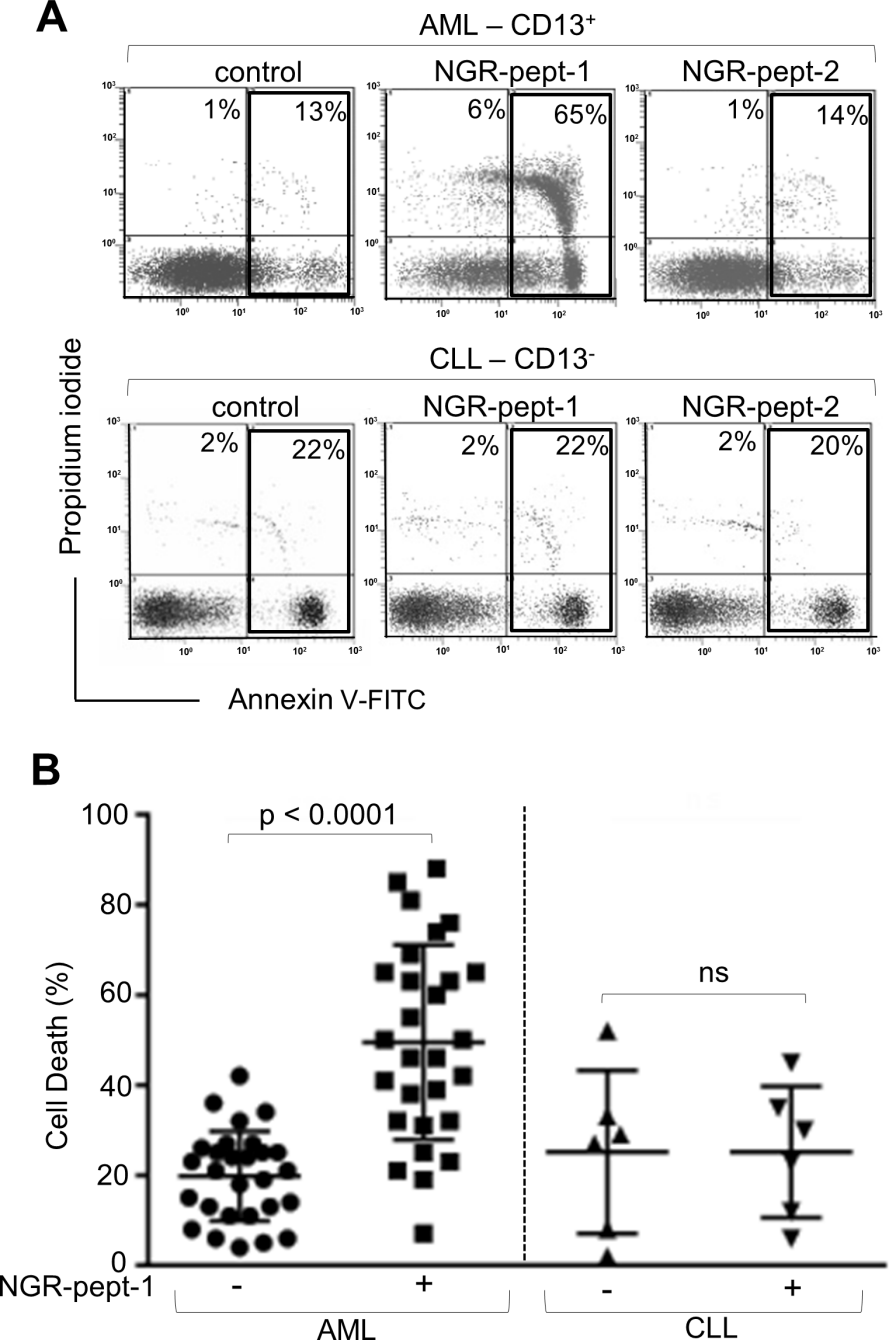
NGR-peptide-1 induces death in primary AML cells (**A, B**) Leukemic blood samples were obtained from 28 AML patients and 6 CLL patients. Isolated PBMCs (10^6^/ml) were cultured in the presence or absence of NGR-peptides (75 μM) for 18 h, stained with annexin-V-FITC/PI and then analyzed by flow cytometry to assess the proportion of dead cells (L2 + L3 gates) in untreated cultures (baseline death) and NGR-peptide-treated cultures. (A) Representative histograms for AML and CLL samples are shown. The proportion (%) of dead cells (L2 + L3 gates) is shown in the box. (B) The proportion (%) of dead cells before and after treatment with NGR-peptide-1. The mean proportion of dead cells is indicated by a horizontal line. Values correspond to the mean ± SD, *P* < 0.0001 compared with non-treated cells, based on a paired Student's *t*-test. ns: non-significant.

The anti-CD13 mAbs WM15 and SJ1D1 were found to bind to surface CD13 on primary blood AML cells but not on cells from patients with chronic lymphocytic leukaemia (CLL) [20, 33]. Leukemic AML and CLL cells were exposed *ex vivo* to NGR-peptides (10–75 μM), and cell death was assessed by the deterioration of cell morphology and PS exposure at various time points. Initial studies performed on 7 AML cell samples showed that NGR-peptide-1's lethal effects occurred in a dose- (≥ 30–75 μM) and time- (≥ 60 min-18 h) dependent manner in 5 AML specimens tested whereas 2 AML samples were resistant to NGR-peptide-1 treatment - even at 75 μM concentration with 18 h or longer incubation times. Consequently, the dose of 75 μM NGR-peptide-1 at 18 h was investigated in all subsequent AML samples. In summary, NGR-peptide-1 induced cell death (≥ 10%) in 24 of the 28 AML samples tested, and this effect was observed in all the French American British (FAB) subtypes tested (Figure 4B). In contrast, NGR-peptide-1 did not affect the viability of 6 CLL cell samples - even with 18 h of treatment at 75 μM (Figure 4A and 4B). It therefore appears that NGR-peptide-1's lethal effect is specific for CD13-positive primary AML cells.

### Treatment with NGR-peptide-1 induces mitochondrial membrane depolarization but not DNA fragmentation

**Figure 5:**
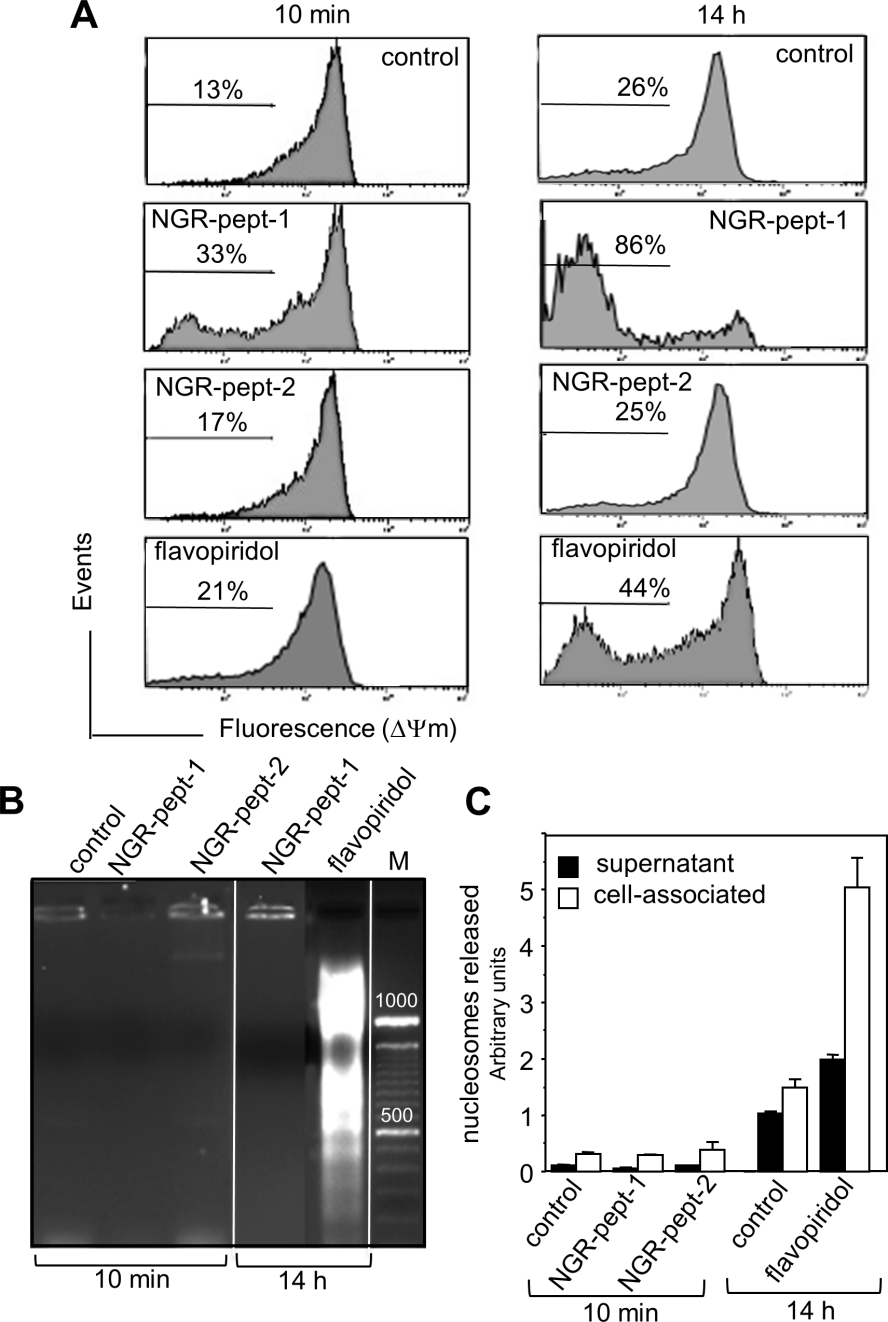
NGR-peptide-1 induces mitochondrial membrane depolarization in the absence of DNA fragmentation (**A–C**) U937 cells were cultured for 10 min and/or 14 h in the absence or presence of 50 μM NGR-peptides or 100 nM flavopiridol (positive control). (A) Then, cells were labelled with the fluorescent probe JC-1. The loss of mitochondrial membrane potential (ΔΨm) is characterized by a significant shift from red (polarization) fluorescence to green (depolarization) fluorescence. Diluted DMSO (corresponding to 100 nM flavopiridol) had no effect on ΔΨm. The percentages refer to ΔΨm dissipation. DNA fragmentation was evaluated by (B) the detection of an oligonucleosome ladder by agarose gel electrophoresis and (C) release of histone-associated DNA fragments (mono- and oligonucleosomes). Data are mean ± SD of three separate determinations.

In further experiments, we therefore decided to investigate the molecular mechanisms underlying the death induced by NGR-peptide-1 in U937 cells. We first sought to determine whether or not NGR-peptide-1 could elicit the mitochondrial and nuclear components of cell death. Flavopiridol was used as a positive control for cell death, since it activates the intrinsic pathway of U937 cell death with disruption of the mitochondrial transmembrane potential (ΔΨm), caspase activation and DNA fragmentation [34–37]. In a fluorescence-based assay, the exposure of cells to NGR-peptide-1 (50 μM) for 10 min or 14 h resulted in dissipation of the ΔΨm by 33% and 86% respectively (evaluated as a decrease in fluorescence intensity, relative to non-treated or NGR-peptide-2-treated cells; Figure 5A). As expected, flavopiridol (0.1 μM) treatment resulted in dissipation of the ΔΨm (by 21% at 10 min and 44% at 14 h) (Figure 5A). The decrease in ΔΨm proceeded in parallel with the increase in cell death. As previously described [38], DNA fragmentation (evaluated as the detection of an oligonucleosome ladder in agarose gel electrophoresis) was evidenced in flavopiridol-treated U937 cells (14 h) (Figure 5B) and was confirmed by a nucleosome detection ELISA (which detects histone-associated mono- and oligonucleosomes in cell culture supernatants and cytoplasmic preparations) (Figure 5C). In contrast, DNA fragmentation (at 10 min and 14 h) was not observed in NGR-peptide-1-treated U937 cells or their culture supernatants (Figure 5B and 5C). The features of NGR-peptide-1-induced ΔΨm disruption and DNA fragmentation were similarly observed in HL- 60, NB4 and THP-1 cells (data not shown). These results show that NGR-peptide-1 induces a cell death process associated with disruption of ΔΨm but not with nuclear fragmentation.

### NGR-peptide-1-induced cell death does not depend upon the Bcl-2 family proteins and the activity of caspases

**Figure 6:**
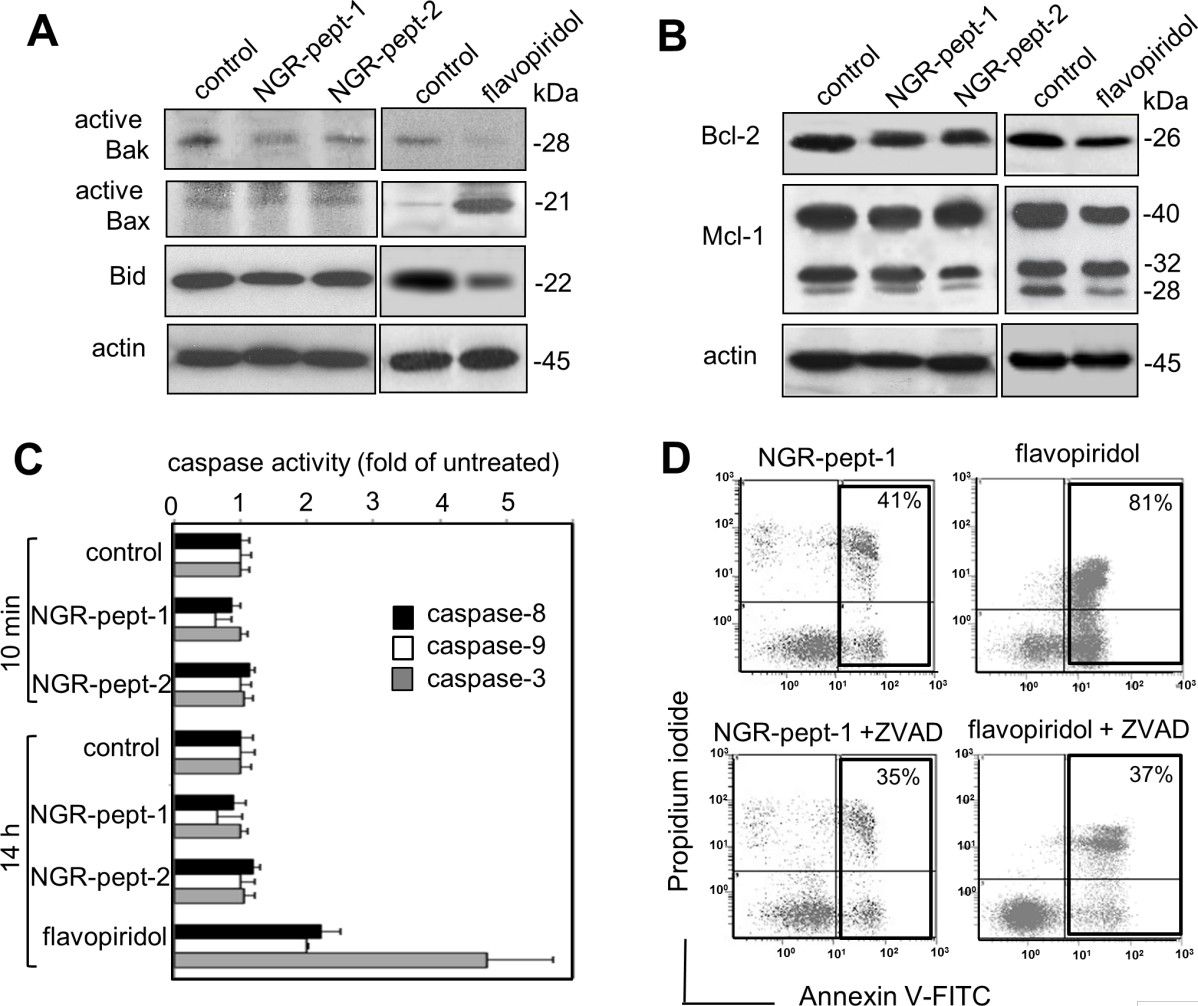
NGR-peptide-1-induced cell death does not depend upon Bcl-2 family proteins and caspases' activity (**A** and **B**) U937 cells were cultured for in the absence or presence of 50 μM NGR-peptides for 10 min or 100 nM flavopiridol for 14 h (positive control) or diluted DMSO (control for flavopiridol). After which lysates were western blotted with antibodies against (A) the pro-apoptotic proteins Bak and Bax (active proteins) and Bid, (B) the anti-apoptotic proteins Bcl-2 and Mcl-1, and (A, B) actin. One of three representative experiments is shown. (**C**) U937 cells were cultured for in the absence or presence of 50 μM NGR-peptides for 10 min and 14 h, or 100 nM flavopiridol for 14 h. Caspase-3,-8 and -9 activities were determined using the substrates DEVD-pNA, IETD-pNA and LEHD-pNA, respectively. Release of pNA was measured at 405 nm. Data are expressed as a fold-increase relative to the corresponding untreated samples (baseline values for caspase-8, caspase-9, and caspase-3 activity at 10 min were 17 ± 2, 4 ± 1, and 8 ± 2 pmol/60 min/mg protein at 37°C, respectively; baseline values for caspase-8, caspase-9, and caspase-3 activity at 14 h were 21 ± 2, 6 ± 2, and 12 ± 2 pmol/60 min/mg protein at 37°C, respectively). Data are mean ± SD of three independent determinations. (**D**) Cells were incubated with 50 μM NGR-peptides for 15 min or 100 nM flavopiridol for 14 h, after 30 min of pretreatment with 50 μM ZVAD-fmk (a broad-spectrum caspase inhibitor). The proportion (%) of dead cells (L2 + L3 gates) is shown in the box. No effect was observed with ZVAD-fmk alone. One of three representative experiments is shown.

ΔΨm can sometimes be disrupted through the action of Bcl-2 family proteins [39]. In particular, the arrangement of the pro-apoptotic proteins Bax and Bak in mitochondrial membrane-bound complexes has a critical role in rapidly permeabilizing the mitochondrial outer membrane [40]. Activation of Bax and Bak depends upon on the balance between anti-apoptotic Bcl-2 proteins (such as Bcl-2 and Mcl-1) and pro-apoptotic BH3-only proteins (such as truncated Bid) [40]. Mcl-1 is a labile protein subject to rapid degradation via several pathways [41]. Bid is a known caspase-8 substrate, and the subsequent production of truncated Bid can activate the mitochondrial pathway of cell death [42]. Non-treated U937 cells expressed low baseline levels of active Bak pro-apoptotic protein (Figure 6A) and high levels of Bcl-2 and Mcl-1 anti-apoptotic proteins (Figure 5B). Furthermore, non-treated U937 cells expressed very low baseline levels of active Bax pro-apoptotic protein (Figure 6A). In agreement with previous studies of U937 cells [38, 43, 44], 14 h of treatment with 0.1 μM flavopiridol increased relative levels of active Bax and decreased levels of Bcl-2, Mcl-1 and Bid (Figure 6A and 6B). After 10 min of cell culture with 50 μM NGR-peptides-1 or -2, no changes in the levels of any of these proteins were observed (Figure 6A and 6B). Non-treated U937 cells expressed high levels of Bid, and NGR-peptide-1 treatment did not lead to either loss of intact Bid (Figure 6A) or formation of tBid fragments (14–15 kDa) (data not shown).

The Bcl-2 members regulate cell death pathways that result in the activation of caspases-9,-8 and -3 and caspase-activated DNase, which leads to nuclear DNA fragmentation [45, 46]. Here, we measured caspase levels in a chromogenic enzyme assay. Non-treated U937 cells displayed detectable baseline levels of all three caspase activities. Flavopiridol (0.1 μM, for 14 h) triggered U937 cell death by activating the caspases (Figure 6C) [36]. In contrast, cell treatment with NGR-peptide-1 (50 μM) for 10 min and 14 h did not affect caspase activities, relative to non-treated cells or NGR-peptide-2-treated cells (Figure 6C). Accordingly, the broad-spectrum caspase inhibitor Z-VAD-fmk inhibited flavopiridol-mediated apoptosis but did not block NGR-peptide-1-mediated cell death (Figure 6D). These results indicate that NGR-peptide-1 induces mitochondrial-dependent cell death through a mechanism independent of Bcl-2 proteins and caspases.

### NGR-peptide-1-induced cell death involves calcium influx and superoxide anion production

**Figure 7:**
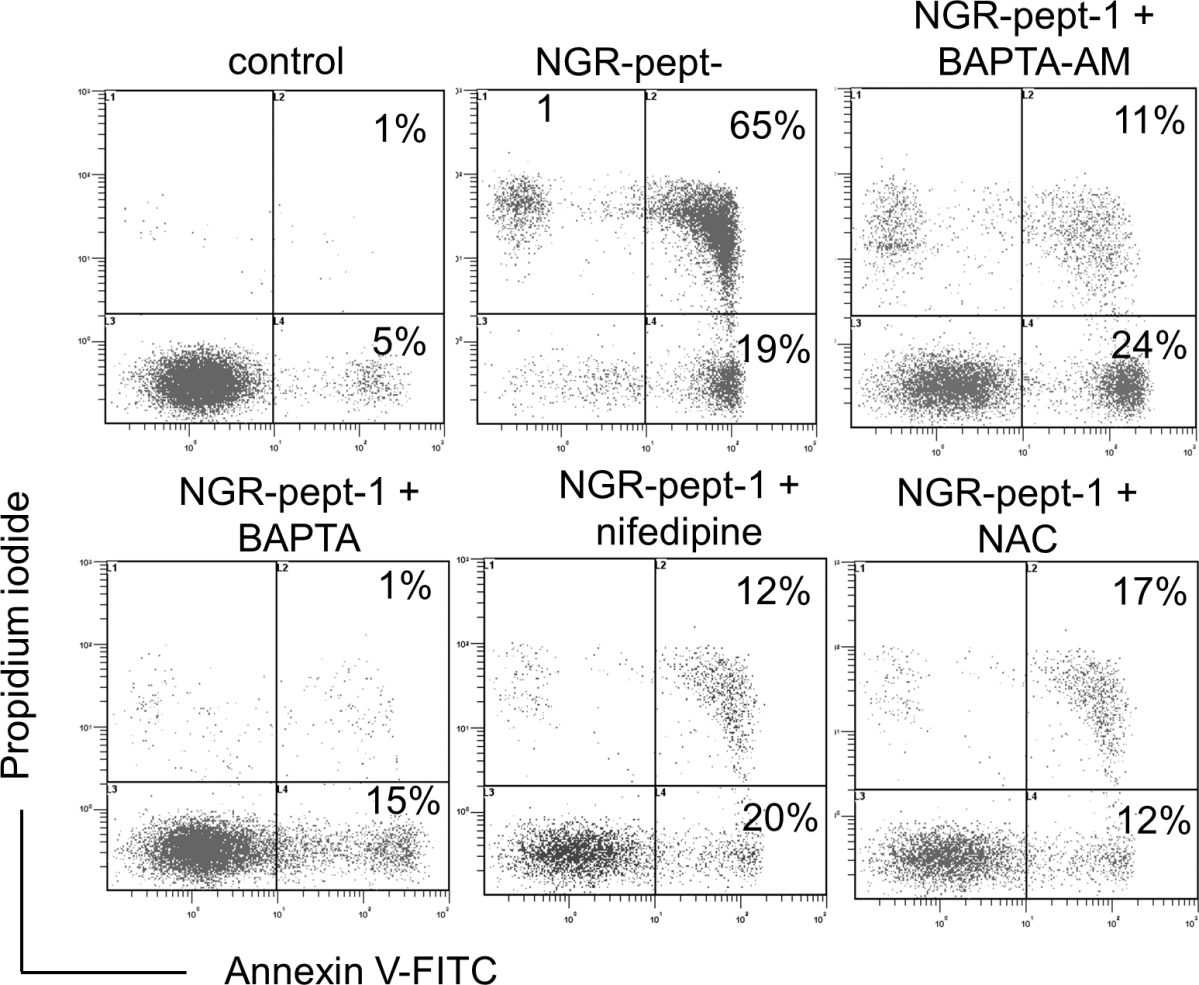
BAPTAs, nifedipine and NAC prevent NGR-peptide-1-induced U937 cell death U937 cells were incubated in the absence or presence of 50 μM NGR-peptides for 15 min, after 30 min of pretreatment with NAC (1 mM) or nifedipine (0.1 mM) or BAPTA (1 mM) or BAPTA-AM (1 mM) or diluted DMSO (control of BAPTA-AM). The proportion (%) of dead cells (L2 and L3 gates) is shown in the box. No effect was observed with BAPTAs, NAC, nifedipine or DMSO (vehicle of BAPTA-AM) alone. One of three representative experiments is shown.

Mitochondrial Ca^2+^ uptake can lead to mitochondrial dysfunction, with ΔΨm depolarization, the release of apoptogenic proteins and/or the production of reactive oxygen species (ROS), leading to cell death [47–49]. We therefore investigated the possible relationships between the NGR-peptide-1 lethal effects, Ca^2+^ release and ROS production in U937 cells. Firstly, we analyzed the ability of two Ca^2+^ chelators (the cell-impermeant compound BAPTA and the cell-permeant compound BAPTA-AM) and nifedipine (known to block L-type Ca^2+^ channels in U937 cells [50]) to modulate NGR-peptide-1-induced cell death (as determined by annexin-V-FITC/PI staining). In absence of NGR-peptide-1, these inhibitors did not alter surface CD13 levels. The chelation of intracellular Ca^2+^ by BAPTA-AM resulted in strong inhibition of NGR-peptide-1-mediated cell death (Figure 7). In fact, cell death appeared to be due to the influx of Ca^2+^ from the extracellular medium, since NGR-peptide-1 was unable to induce cell death when extracellular Ca^2+^ was chelated by cell-impermeant BAPTA (Figure 7). Nifedipine also protected the cells against death triggered by NGR-peptide-1 (Figure 7). This finding also implies that NGR-peptide-1 triggers cell death by inducing extracellular Ca^2+^ entry through L-type Ca^2+^ channels.

**Figure 8:**
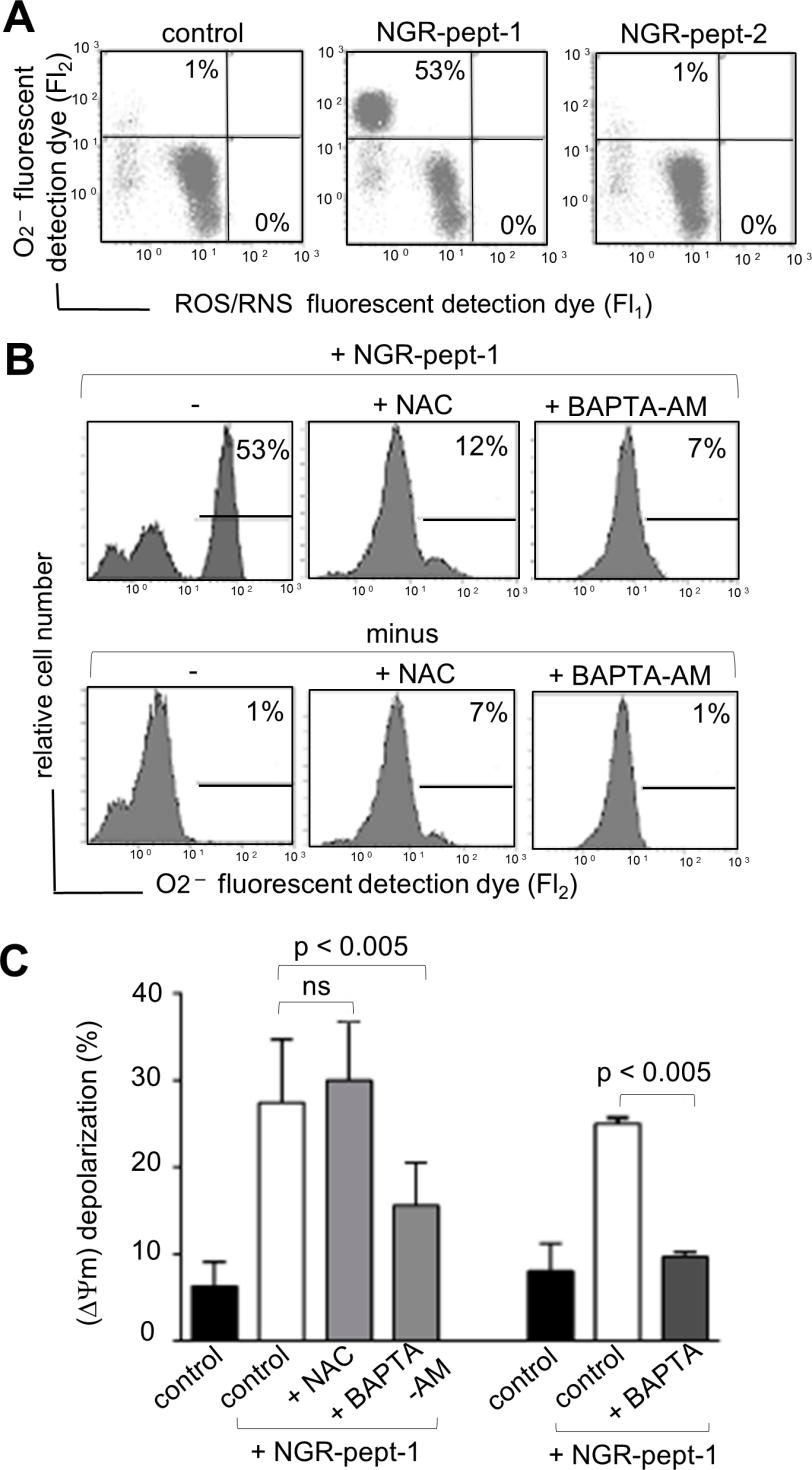
Effects of BAPTAs and NAC on ROS/RNS production and ΔΨm depolarization in NGR-peptide-1-treated U937 cells U937 cells were incubated in the absence or presence of 50 μM NGR-peptide-1 for 10 min, after 30 min of pretreatment with BAPTA, BAPTA-AM or NAC (1 mM) or diluted DMSO (control for BAPTA-AM). (**A, B**) Cells were labelled simultaneously with two dyes which react respectively with superoxide anion (O_2_^−^) (generating an FL2 fluorescent product) and other ROS/RNS types (H_2_O_2_, ONOO, HO·, NO and ROO·) (generating an FL1 fluorescent product). Production of ROS/RNS and O_2_^−^ is highlighted in the corresponding box and their percentage is shown in the Figure. One representative experiment is shown. Experiments were repeated at least three times. (A) Effects of NGR-peptides on ROS/RNS and O_2_^−^ production. (B) Effects of BAPTA-AM and NAC on ROS/RNS and O_2_^−^ production by NGR-peptide-1 treated cells. (**C**) Effects of BAPTAs and NAC on ΔΨm depolarization in non-treated and NGR-peptide-1 treated cells. No effect was observed with BAPTAs, NAC, or DMSO (vehicle of BAPTA-AM) alone. Data are mean ± SD of four independent experiments. *P* < 0.005 compared with NGR-peptide-1-treated cells, based on an ANOVA.

Moreover, the induction of cell death by NGR-peptide-1 was blocked by a 30-minute pretreatment with the antioxidant N-acetylcysteine (NAC, 1 mM) (Figure 7). We therefore analyzed intracellular levels of ROS in NGR-peptide-1-treated cells. Cells were labelled simultaneously with two fluorescent dyes that react respectively with superoxide anion (O_2_^−^) only (giving a FL2 product) and with other types of ROS/reactive nitrogen species (RNS) (H_2_O_2_, ONOO^−^, HO·, NO and ROO·) (giving a FL1 product). As shown in Figure 8A, a 10-minute treatment with NGR-peptide-1 induced the production of O_2_^−^ but not of other types of ROS/RNS, when compared with NGR-peptide-2 treatment or the absence of treatment. The addition of NAC (1 mM) prevented the generation of O_2_^−^ in NGR-peptide-1-treated cells (Figure 8B). Moreover, the effect of NGR-peptide-1 on O_2_^−^ production was also abrogated with BAPTA-AM (Figure 8B) - indicating that intracellular Ca^2+^ has a key role in O_2_^−^ generation by NGR-peptide-1-treated cells. The production of O_2_^−^ proceeded almost in parallel with the decrease in ΔΨm. As seen for the inhibition of the O_2_^−^ production, BAPTA inhibitors also inhibited ΔΨm disruption (Figure 8C). However, NGR-peptide-1 treatment with NAC failed to prevent ΔΨm dissipation (Figure 8C) indicating that the O_2_^−^ produced is not involved in ΔΨm depolarization. Taken as a whole, our data indicate that cell death induced by NGR-peptide-1 involves the influx of extracellular Ca^2+^, Ca^2+^-mediated ΔΨm disruption and mitochondrial O_2_^−^ generation.

### NGR-peptide-1 induces 88 kDa progranulin degradation - possibly via the activation of promatrix metalloproteinase-12 (proMMP-12) by O_2_^−^

**Figure 9:**
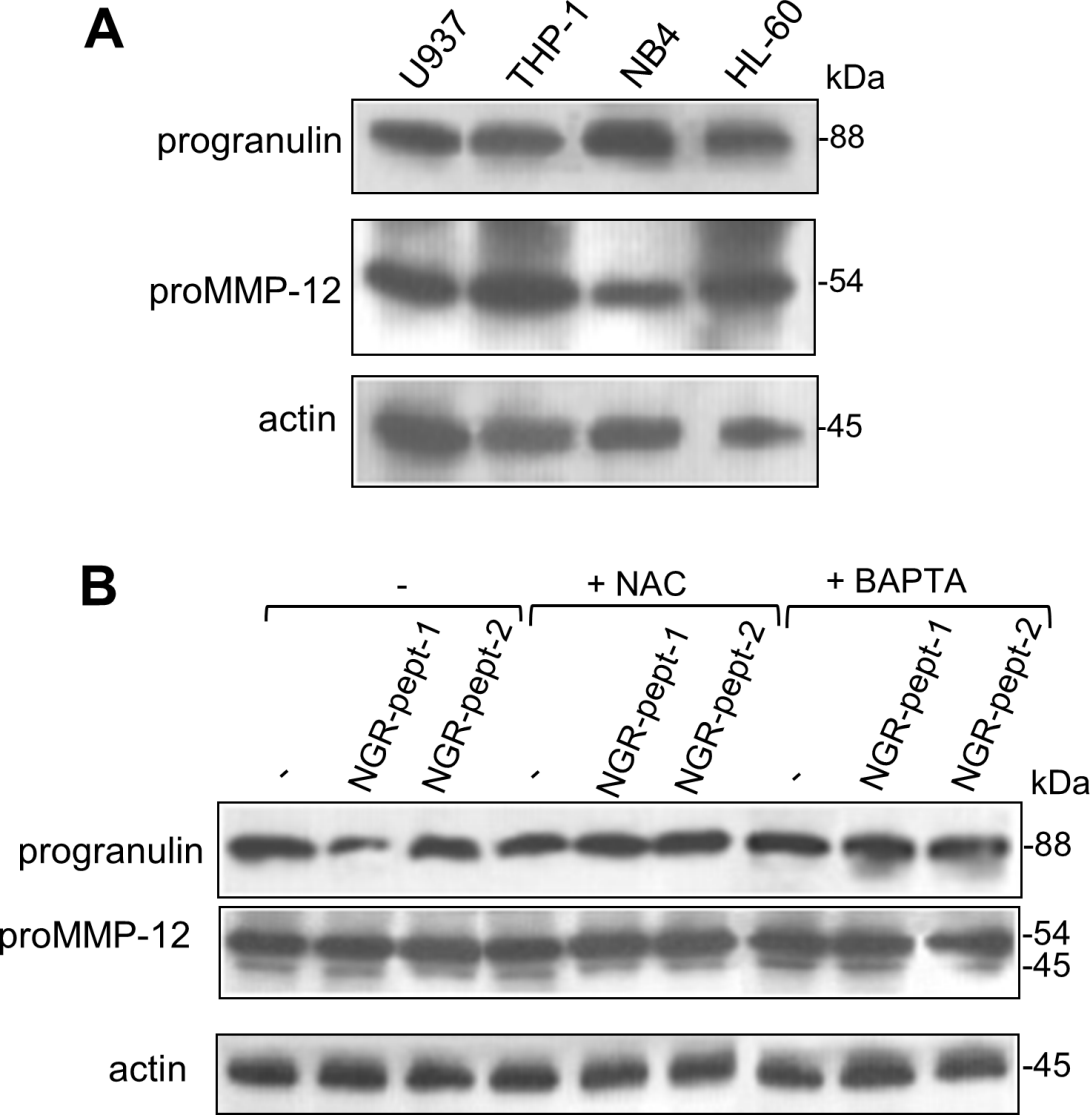
Expression of progranulin and proMMP-12 in AML cell lines and effect of NGR-peptide-1 on their expression in U937 cells (**A**) Cell lysates from HL-60, THP-1, U937 and NB4 cells were examined for progranulin, proMMP-12 and actin expression in an immunoblot assay. (**B**) U937 cells were treated for 10 min with 50 μM NGR-peptides after a 30 min pretreatment with 1 mM BAPTA or NAC. Lysates were then western blotted with antibodies against progranulin, MMP-12 (active and latent forms) and actin. One of three representative experiments is shown.

There is a growing body of evidence to suggest that ROS can activate proMMPs via the oxidation of cysteine residues in the MMP prodomain [51]. ProMMP-12 is mainly produced by myeloid cells [52]. The 88 kDa MMP- 12 substrate progranulin [53] is a known cell survival factor [54, 55]. Progranulin inactivation via its degradation has been linked to cell death [54, 55]. We therefore hypothesized that O_2_^−^ generated as a result of NGR-peptide-1 treatment can activate proMMP-12, which in turn could degrade progranulin. As previously described for U937 cells [56, 57], non-treated AML cell lines expressed proMMP-12 (54 kDa) and progranulin (88 kDa) proteins (Figure 9A). After 10 min of cell culture with 50 μM NGR-peptides, the amount of progranulin protein was markedly lower in NGR-peptide-1-treated cells than in NGR-peptide-2-treated and non-treated cells (Figure 9B for U937 cells and data not shown for the other cell lines); we did not observed any of the 45–12 kDa progranulin fragments previously described [53]. BAPTA and NAC blocked the decrease in progranulin levels (Figure 9B). In all cases, the levels of proMMP-12 were similar to control values (Figure 9B).

**Figure 10:**
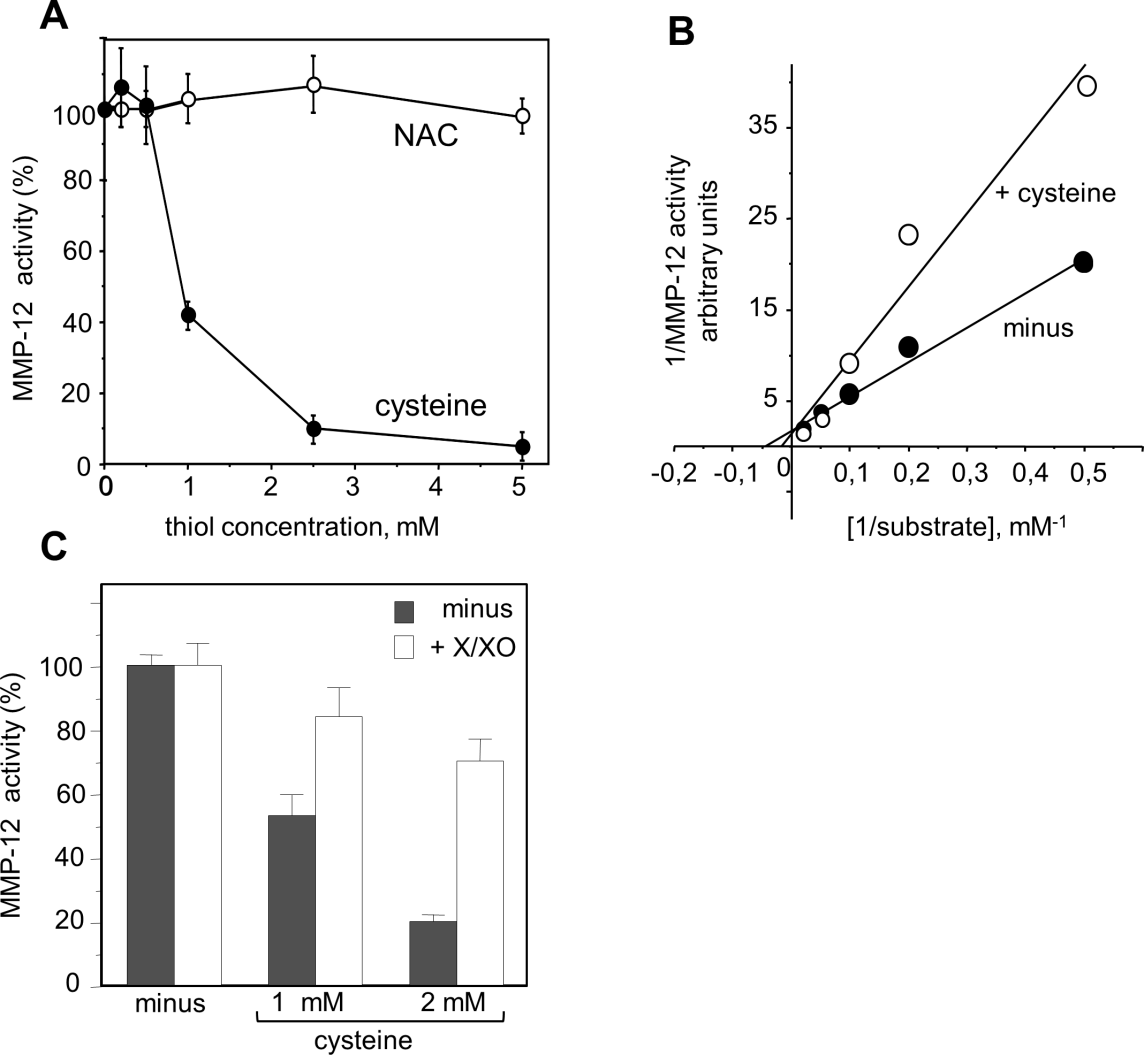
Redox sensitivity of MMP-12 activity Hydrolytic activity of active recombinant MMP-12 was determined by measuring the fluorescence released by cleavage of the substrate Mca-Pro-Leu-Ala-Gln-Ala-Val-Dpa-Arg-Ser-Ser-Ser-ArgNH_2_. (**A**) MMP-12 activity was measured in the absence or the presence of increasing concentrations of cysteine or NAC (0.2–5 mM). (**B**) The K_m_ and V_max_ were calculated from a Lineweaver-Burk plot. The Km in the absence and presence of cysteine were 20 μM and 50 μM respectively. (**C**) MMP- 12 activity was measured in the absence or the presence of cysteine or NAC (1 and 2 mM) and/or enzymatic O_2_^−^ -generating system (100 μM xanthine and 0.1 U/ml xanthine oxidase). The data are expressed as a percentage, relative to non-treated cells. The data are quoted as the mean ± SD from three determinations.

We therefore looked at whether or not proMMP-12 activation by O_2_^−^ was involved in NGR-peptide-1-mediated progranulin cleavage during AML cell death. Levels of endogenous MMP-12 activity were similar in NGR-peptide-1-treated, NGR-peptide-2-treated and non-treated U937 cell lysates; this was probably due to the spontaneous activation of proMMP-12 by the detergent used to lyse cells [58]. We therefore used an *in vitro* assay to determine the effects of redox conditions on the catalytic activity of active, recombinant MMP-12. We found that cysteine (which mimics the prodomain's cysteine) dose-dependently inhibited MMP-12 activity, whereas NAC did not (Figure 10A). The maximal reaction rates (Vmax) in the absence and presence of 1 mM cysteine were similar (Figure 10B), indicating that cysteine interferes with MMP- 12's catalytic site. Cysteine-mediated inhibition of MMP-12 activity was blocked by generation of O_2_^−^ via xanthine/xanthine oxidase treatment (Figure 10C). Taken as a whole, these data suggest that O_2_^−^ (possibly through proMMP-12 activation) is linked to 88 kDa progranulin cleavage during NGR-peptide-1-mediated death in AML cells.

### A distinct 105 kDa progranulin isoform correlates to AML blast resistance to NGR-peptide-1

**Figure 11:**
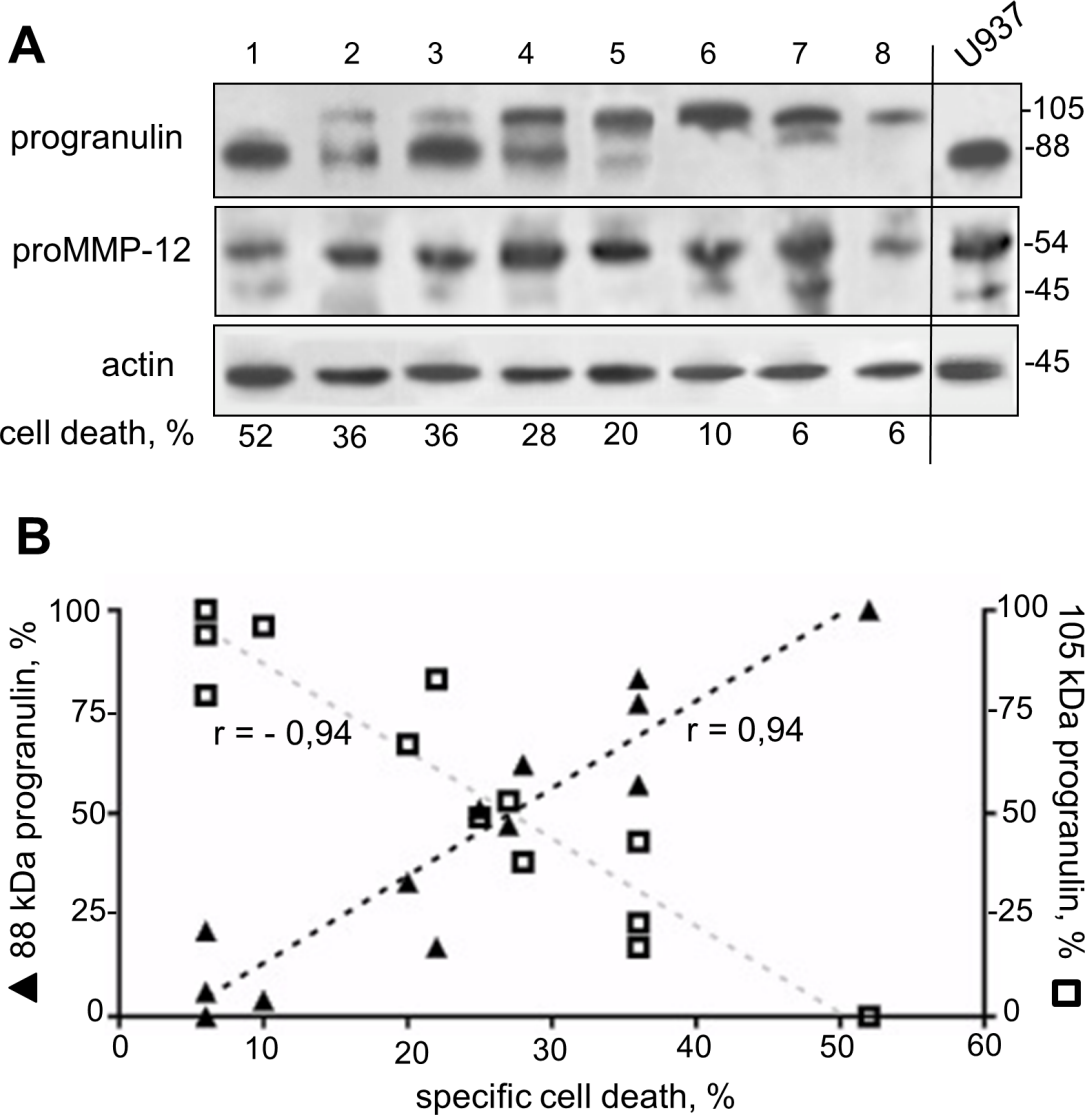
Expression of a distinct 105 kDa progranulin isoform in AML specimens Cell lysates from 13 AML blood samples were examined for progranulin, proMMP-12 and actin expression in immunoblot assays. (**A**) Representative blots (8 AML and control U937) are shown. (**B**) Expression of 88 kDa and 105 kDa progranulin isoforms as a function of specific NGR-peptide-1-mediated cell death which was obtained by subtracting the percentage of basal death in untreated cells from the percentage of death in the corresponding NGR-peptide-1-treated cells. *n* = 13, Pearson r correlation *P* < 0.0001.

We retrospectively analyzed the expression profiles of progranulin and proMMP-12 proteins in 13 primary AML specimens sensitive or resistant to the lethal effect of NGR-peptide-1. Unexpectedly, the anti-progranulin Ab detected the 88 kDa progranulin as well as a distinct 105 kDa progranulin isoform (Figure 11A): AML patient samples (*n* = 9) in which NGR-peptide-1 induced cell death (> 20%) expressed both isoforms or the 88 kDa isoform alone (Figure 11A), whereas NGR-peptide-1-resistant samples (*n* = 4) preferentially expressed the 105 kDa isoform (Figure 11A). Indeed, there was a positive correlation between 88 kDa progranulin expression and marked cell death in NGR-peptide-1-treated AML blasts (Figure 11B); conversely, the expression of the 105 kDa isoform was associated with AML blast resistance to NGR-peptide-1 (Figure 11B). The protein band corresponding to proMMP-12 was seen in all primary AML samples (Figure 11A). These data suggest that the expression of a distinct, 105 kDa progranulin isoform in AML blasts is associated with AML blast resistance to NGR-peptide.

## DISCUSSION

Our results show that CNGRC-GG-_D_(KLAKLAK)_2_ (NGR-peptide-1) kills AML primary cells and AML cell lines by targeting leukemic surface CD13. We demonstrated that the peptide induces a caspase-independent programmed cell death by recruiting two importance molecular messengers in cancer cell death: extracellular Ca^2^ and mitochondrial O_2_^−^.

The AML cell lines (U937, HL-60, NB4 and THP-1) rapidly underwent death after CNGRC-GG-_D_(KLAKLAK)_2_ (NGR-peptide-1) treatment (as evidenced by PS externalization and cell shrinkage), whereas CNGRC-G, _D_(KLAKLAK)_2_ (free peptide-1) and CNGRC-GG-LVTT did not affect cell viability, thus indicating that the lethal action of NGR-peptide-1 is both dependent on CD13 targeting and _D_(KLAKLAK)_2_. Our results in AML cell lines were confirmed by experiments on primary blood AML cells. It remains to be seen whether NGR-peptide-1 can kill leukemic cells from AML patients who do not respond to treatment or have developed chemoresistance. Our data disagree with previous suggestions that the NGR-targeted drug approach is not necessarily valid in myeloid cells - based on the CRNGRGPDC peptide's failure to bind to U937 cells [59] and the CNGRCG-TNF-α peptide's failure to bind to THP-1 cells [16].

We next investigated the signalling pathways underlying NGR-peptide-1-induced AML U937 cell death. Caspase-dependent apoptotic processes can be achieved through death receptors (in the extrinsic pathway) or the mitochondria (in the intrinsic pathway) [46, 60]. The Bcl-2 family members are involved in these cell death pathways via the activation of caspases and then nuclear DNA fragmentation by caspase-activated DNase [45, 46, 61]. In the present study, NGR-peptide-1 was found to disrupt ΔΨm but did not modulate Bcl-2 pro-apoptotic proteins (i.e. Bax, Bak, Bid), Bcl-2 anti-apoptotic proteins (i.e. Bcl-2 and Mcl-1), caspase (−3, −8, −9) activation or nuclear DNA fragmentation. These data therefore indicate that NGR-peptide-1 does not induce AML cell death through the intrinsic and extrinsic caspase-dependent apoptotic pathways.

The pattern of NGR-peptide-1-mediated AML cell death resembles characteristics of caspase-independent necrosis [62] but appears distinct of paraptosis [63]. Indeed, while paraptosis is blocked by cycloheximide [63], U937 cell death induced by NGR-peptide-1 was not, thus suggesting merely a mode of regulated necrosis. Various types of cell death (referred to as regulated necrosis) with morphological similarities to necrosis have already been identified including necroptosis, parthanatos and mitochondrial permeability transition (MPT)-dependent regulated necrosis [60, 64]. NGR-peptide-1-mediated cell death does not exhibit the characteristics of necroptosis (defined by caspase-8 activity and cell death inhibition by Z-VAD-fmk) and parthanatos (defined by DNA damage) [60]. A large variety of molecules and processes have been characterized as initiators, modulators or effectors of regulated necrosis [60, 64]. Free Ca^2+^ stimulates the opening of the MPT pore, with the ensuing release of apoptogenic proteins and ROS into the cytosol [47–49]; this may in turn contribute to the induction and execution of regulated necrosis [64]. The superoxide anion (O_2_^−^) is the main ROS generated by the mitochondrial electron-transport chain [51, 65]. A few *in vitro* studies have highlighted the lethal effects of mitochondrial O_2_^−^ in human tumour cells (including leukemia cells) [66–68]. The results of our experiment with Ca^2+^ chelators and the Ca^2+^ channel blocker nifedipine demonstrate that NGR-peptide-1 induces cell death through the influx of extracellular Ca^2+^, which in turn leads to ΔΨm disruption. Moreover, NGR-peptide-1 treatment specifically causes O_2_^−^ generation; the latter is blocked by the antioxidant NAC and the Ca^2+^ chelator BAPTA - demonstrating that O_2_^−^ acts as a second, critical signal in NGR-peptide-1-mediated cell death. Although NAC almost totally prevented O_2_^−^ production and death, it did not affect ΔΨm depolarization. This finding indicates that O_2_^−^ is generated after ΔΨm depolarization (probably by the mitochondria). Taken as a whole, our observations convincingly show that NGR-peptide-1-induces a mode of regulated necrosis through the Ca^2+^-mitochondrial O_2_^−^ pathway.

We further analyzed the mechanisms by which NGR-peptide-1 induced the entry of extracellular Ca^2+^. As evoked above, NGR-peptide-1 triggers cell death by enhancing Ca^2+^ entry through L-type channels. The Ca^2+^ influx is associated with surface CD13 downregulation in NGR-peptide-1-treated cells, which probably reflects CD13 endocytosis. The fact that NGR-peptide-2 (the negative control) does not induce surface CD13 decrease strongly suggests that peptide-1 (when conjugated to NGR) actively participates in the downregulation of CD13. Cationic peptides such as peptide-1 (_D_(KLAKLAK)_2_), by interacting with and permeating membrane's anionic phospholipids [23, 69], may disturb the normal bilayer structure of the plasma membrane [70]. Endocytic pathways require lipid rafts, which are enriched in cholesterol, glycosphingolipids, various receptors, membrane transporters and signal-transducing kinases [71]. L-type Ca^2+^ channels are present in lipid rafts [71]. Similarly, CD13 is associated with lipid rafts in myeloid cells [72]. The Ca^2+^ signalling has been further demonstrated to initiate from the lipid rafts [71]. One can therefore legitimately hypothesize that in AML cells, CD13 and Ca^2+^ channels are co-expressed in resting state rafts. One can imagine that by binding to surface CD13, NGR-peptide-1 modifies the conformation of CD13 leading to a destabilization of the cell membrane, favoring the interaction of peptide-1 with the bilayer of phospholipids, which in turn opens Ca^2+^ channels in lipid rafts and leads to the entry of extracellular Ca^2+^. Although our results reveal the new role of NGR-peptide-1 in Ca^2+^ influx and signalling, two key questions remain: is NGR-peptide-1 endocytosed with CD13? And if so, can it target mitochondria? Corti's group has suggested that NGR-peptide-1's cytotoxic effect on the endothelial KS1767 cell line is based on internalization and binding to mitochondrial anionic phospholipids, resulting in the loss of ΔΨm [9]. In view of our results, it is possible that NGR-peptide-1's lethal effect in KS1767 cells corresponds to a downstream event in the execution of the cell death pathway. Moreover, one can imagine that peptide-1 carried by the other designed tumor homing peptides [24–27] is also capable of disrupting the plasma membrane. These points merit further investigation.

Our data suggest that O_2_^−^ has a critical role in NGR-peptide-1-mediated regulated necrosis in AML cells. What, then, might O_2_^−^'s intracellular targets be? ROS (including O_2_^−^) can oxidize the sulfhydryl groups of proteins, thereby modifying the latter's conformation and functions [51, 73]. For instance, O_2_^−^ has been linked to proteasome activity and the stability of certain anti-apoptotic Bcl-2 family proteins [65]; an O_2_^−^ increase in B lymphocytes triggers apoptosis by favouring the proteasome-mediated degradation of Mcl-1 [74]. In the present study, NGR-peptide-1 did not affect the proteasome's chymotrypsin-like activity in U937 cells (data not shown) or modify protein levels of Bcl-2 and Mcl-1 - indicating that proteasome activity is not involved in NGR-peptide-1's O_2_^−^-mediated lethal action. Other ROS targets include the proMMPs, which are synthesized in a latent, zymogen form [75]. Zymogen conformation and thus latency are maintained by the coordination of Zn^2+^ in the MMP's catalytic domain by a PRCGXPD cysteine switch motif in the prodomain [75]. By disrupting the cysteine-zinc binding, ROS are able to stimulate the proMMPs' enzymatic activity (as already demonstrated for proMMP-1,-2,-7,-8, and -9) [51, 58, 76]. Conversely, thiols inhibit MMP catalytic activity [77, 78]. Using a cell-free assay, we demonstrated the redox-sensitive control of recombinant MMP-12 activity (inhibition by cysteine and activation by O_2_^−^). ProMMP-12 is a 54 kDa proenzyme that is processed into 45 kDa and 22 kDa active forms [53]. Its expression is closely associated with inflammatory diseases and cancers [79]. In the present study, we showed that proMMP-12 is constitutively expressed in AML cell lines and AML blood cells. Hence, proMMP-12 might well undergo O_2_^−^-dependent activation in NGR-peptide-1-treated AML cells.

Active MMP-12 performs various cellular functions, including the degradation of matrix components, the release of cytokines, growth factors and chemokines, and the modulation of cell motility and transcriptional activity [80, 81]. It was recently reported that progranulin is a substrate for MMP-12 [53]. Progranulin is an 88 kDa glycoprotein that acts as a positive regulator of cell proliferation, survival and migration [54, 55]. Elevated progranulin levels are associated with various human tumours [82–86]. For instance, progranulin is a novel, independent predictor of disease progression and overall survival in CLL [86]. Progranulin is widely expressed in mammalian tissues, with particularly high levels in myeloid cells [55]. We showed that AML cell lines express the 88 kDa progranulin; the level of progranulin protein was downregulated in NGR-peptide-1 treated cells, and this decrease was blocked by BAPTA and NAC. Unexpectedly, a distinct progranulin isoform with a 105 kDa size was detected in primary AML blasts. Progranulin displays a heterogeneous pattern of glycosylation. It has been demonstrated that four of progranulin's five potential N-glycosylation consensus sites are indeed glycosylated [87]. Therefore, the 105 kDa progranulin might correspond to a more highly glycosylated protein. Overall, our data indicate a clear relationship between the decrease in levels of the 105 kDa protein, the increase in levels of the 88 kDa protein, and increased cell death in NGR-peptide-1-treated AML blasts. The expression of the 105 kDa progranulin isoform can be therefore considered as a marker of AML blast resistance to NGR-peptide-1. Next studies are warranted to further assess whether the level of 88 kDa progranulin is downregulated in NGR-peptide-1-responsive AML samples. Taken as a whole, our data demonstrate that 88 kDa progranulin is a target of NGR-peptide-1 (possibly through O_2_^−^-mediated proMMP-12 activation) during death in AML cells. One key question that needs to be addressed is progranulin cleavage passive or active to NGR-peptide-1-mediated AML cell death. However, reduced levels of progranulin (using progranulin small interfering RNA) in U937 cells before NGR-peptide-1 treatment already led to a marked increase in cell death (data not shown). Furthermore, it remains to be established whether MMP- 12 siRNA has the ability to suppress at least in part NGR-peptide-1-mediated cell death.

**Figure 12:**
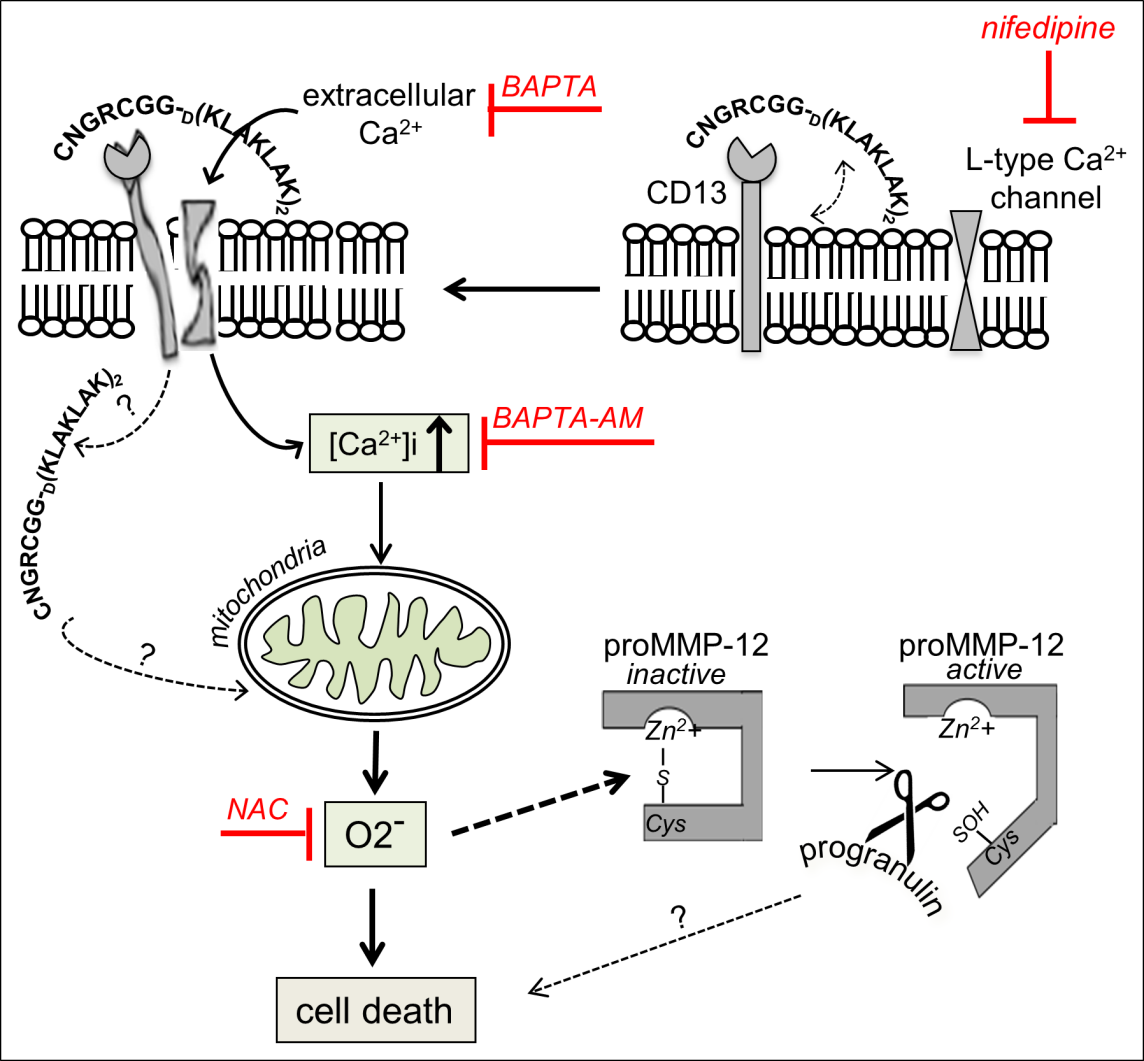
Putative model for the involvement of cell signalling pathways in the induction of death by NGR-peptide-1 in AML cells By binding to surface CD13, CNGRC-GG-_D_(KLAKLAK)_2_ (NGR-peptide-1) could destabilize the plasma membrane, favoring the interaction of peptide-1 with the bilayer of phospholipids, which in turn opens L-type Ca^2+^channels in lipid rafts and leads to the entry of extracellular Ca^2+^. The increase of intracellular Ca^2+^ disrupts ΔΨm, leading to mitochondrial O_2_^−^ release, and induces cell death. Furthermore, NGR-peptide-1 inactivates the 88 kDa survival factor progranulin possibly through proMMP-12 activated by O_2_^−^. The role of progranulin degradation in cell death is not known.

In summary, our results indicate that exposure of AML cells to CNGRC-GG-_D_(KLAKLAK)_2_ elicits a series of related events e.g. Ca^2+^ influx, ΔΨm disruption, mitochondrial O_2_^−^ generation and 88 kDa progranulin inactivation, and a mechanism of action is proposed in Figure 12. AML remains a challenging disease in the clinic because patients are often refractory to front-line therapy or subsequently relapse [18]. A range of drug candidates (including tyrosine kinase inhibitors, farnesyltransferase inhibitors, histone deacetylase inhibitors, multidrug-resistance inhibitors, and deoxyadenosine analogues) is now in clinical development [18, 88]. Interestingly, treatment with Ca^2+^ channel blockers (such as amlodipine or diltiazem) is predictive of worse survival in patients with AML [89]. When administered to mice, NGR-peptide-1 does not induce apparent toxicity and is not immunogenic [9]. The recent review by Zhang *et al.* [90] evaluates the evidence for ROS in eradicating AML stem cells. Therefore, NGR-peptide-1s' ability to promote regulated necrosis via the Ca^2+^/O_2_^−^ pathway may provide a new model for the treatment of AML.

## MATERIALS AND METHODS

### Chemicals and reagents

H-Cys-Asn-Gly-Arg-Cys-Gly-Gly-_D_(Lys-Leu-Ala-Lys-Leu-Ala-Lys-Lys-Leu-Ala-Lys-Leu-Ala-Lys-NH_2_) (disulphide bridge 1–5) [CNGRC-GG-_D_(KLAKLAK)_2_] (NGR-peptide-1), _D_(KLAKLAK)_2_ (peptide-1), CNGRCG (disulphide bridge 1–5) and H-Cys-Asn-Gly-Arg-Cys-Gly-Gly-Leu-Val-Thr-Thr-OH (disulphide bond bridge 1–5) (CNGRCGG-LVTT-OH) (NGR-peptide-2, a negative control) were purchased from Eurogentec (Angers, France) and prepared as 1 mM stock solutions in sterile distilled water. Phycoerythrin (PE)-conjugated anti-CD13 (SJ1D1, mIgG1), PE-mIgG1, anti-CD13 (SJ1D1, mIgG1) and goat F(ab')2 fragment anti-mouse fluorescein isothiocyanate-conjugated Ig (GAM-FITC) were obtained from Beckman-Coulter (Luminy, France). Anti-CD13 (WM15, mIgG1) was purchased from BD-Pharmingen (San Jose, CA, USA). Anti-Bax specific for the active form (6A7, mouse IgG), anti-Bid (FL-195, rabbit IgG), anti-Bcl-2 (100, mouse IgG_1_) and anti-Mcl-1 (S-19, rabbit IgG) antibodies were from Santa-Cruz (Tebu-Bio SA, Le Perray en Yvelines, France). Anti-Bak specific for the active form (TC-100, mouse IgG_2A_) was from Calbiochem (Darmstadt, Germany). Anti-actin (C4, mIgG1) was from ICN Biomedicals (Aurora, OH, USA). The secondary, horseradish peroxidase (HRP)-conjugated antibodies were bought from Dako Cytomation (Glostrup, Denmark). ZVAD-fmk (a general caspase inhibitor), caspase-3/8/9 assay kits, recombinant human proMMP-12 and anti-progranulin (P28799, polyclonal goat Ig) were obtained from R&D Systems (Abingdon, UK). Anti-MMP-12 specific for both the pro- and active forms (AB6010, polyclonal rabbit Ig) was from Millipore (Temecula, CA, USA). The proteasome-Glo™ chymotrypsin-like cell-based assay kit was obtained from Promega-France (Charbonnières-les-Bains, France). Cell-permeant BAPTA-AM (1,2-bis-(2-aminophenoxy)ethane-N,N,N',N'-tetraacetic acid, tetra (acetoxymethyl) ester) was obtained from Life Technologies SAS (Saint Aubin, France). Cysteine, cytochalasin D, nocodazole, nifedipine, cell-impermeant BAPTA (1,2-bis-(2-aminophenoxy)ethane-N,N,N',N'-tetraacetic acid), Succ-Ala- Ala- Val- pNA and flavopiridol were purchased from Sigma (St Louis, MO, USA). Flavopiridol was prepared as a 10 mM stock solution in DMSO. The MMP-12 substrate[7-methoxycoumarin-4-yl)acetyl-Pro-Leu-Ala-Gln-Ala-Val-N-3(2,24-dinitrophenyl) -L-a,b diaminopropionyl]-Arg-Ser-Ser-Ser-ArgNH_2_ was purchased from Enzo Life Sciences (Villeurbanne, France).

### Cells and treatments

Leukemic blood samples from 28 AML patients and 6 chronic lymphocytic leukemia (CLL) patients were obtained from the “*Tumorothèque Hématologie*” biological resource centre at Saint-Antoine Hospital (Paris, France). These samples were collected after approval by the Institutional Review Board and after patients provided informed consent in accordance with the Declaration of Helsinki 2002. The diagnosis of AML was established in accordance with standard clinical criteria and the FAB Committee's cytological criteria: M0/undifferentiated (1 sample), M1/myeloblastic (5 samples), M2/myeloblastic with maturation (5 samples), M4/myelomonocytic (6 samples) and M5/monoblastic (11 samples). CLL (Binet stage A) was diagnosed according to standard clinical criteria and the guidelines issued by the International Workshop on CLL. Peripheral blood mononuclear cells (PBMCs) were separated by Ficoll-Hypaque density gradient (1.077 g/ ml) centrifugation. AML cells were CD13^+^CD33^+^. CLL cells were CD5^+^CD20^+^CD23^+^CD13^−^. Leukemic cells (10^6^/ ml) were cultured in RPMI 1640 medium supplemented with 10% heat-inactivated foetal calf serum (FCS) (LPS < 0.1 ng/ ml), 2 mM L-glutamine, 1 mM sodium pyruvate and 40 mg/ml gentamycin (Gibco) in a 5% CO_2_ humidified atmosphere at 37°C. The AML cell lines U937 (CRL-1593.2; FAB M5), THP-1 (202-TIB; FAB M5) and HL-60 (240-CCL; FAB M2) cells were purchased from American Type Culture Collection (ATCC). NB4 cells (FAB M3) were obtained from Dr. Michel Lanotte's laboratory (Hôpital Saint-Louis, Paris, France) [91]. All cell lines were phenotyped (for CD11b, CD11c, CD15, CD44, CD13), tested for mycoplasm every 4 months, and used within 3–10 passagings. The induction of differentiation of NB4 and HL-60 cells into granulocyte-like cells by all-trans retinoic acid (increase of CD15 in HL-60 and NB4 cells, and the downregulation of CD13 and CD44 in HL-60 cells) and the induction of differentiation of all cell lines into macrophage-like cells (increase of CD11b and CD11c) by phorbol myristate acetate were monitored every 4 months. Cells were cultured in complete RPMI 1640 medium supplemented with 5% FCS or 10% (THP- 1) in a 5% CO2 humidified atmosphere at 37°C. For all experiments, proliferating U937 cells were harvested in the log-phase at passage 12 or less. Cells (2 × 10^5^/ml) were treated with NGR-peptides (1–100 μM) for various periods of time. Flavopiridol (0.1 μM) was used as a positive control for cell death induction. In negative control experiments, cells were treated with the same volume of water or DMSO (the vehicles for peptides and flavopiridol, respectively). The inhibitors (such as NAC, BAPTAs and nifedipine) were added 30 min before addition of NGR-peptides or flavopiridol.

### Cell viability and morphology

The number of viable cells (with diameters ranging from 9 to 14 μM) and dead cells (with diameters ranging from 4 to 9 μM) was counted with a Coulter ZM_2_ (Beckman-Coulter, Villepinte, France). The U937 cells' morphology was assessed by light microscopy of cytocentrifuged cells stained with the Hemacolor kit from Merck.

### The annexin-V/PI staining assay

Cell death was assessed by using the detection cell death kit (Beckman-Coulter), according to the manufacturer's instructions. Phosphatidylserine externalization was quantified by the specific binding of FITC-conjugated annexin-V and simultaneous labelling with PI (reflecting cell membrane disruption during cell death). Stained cells (20,000) were analyzed with a flow cytometer (Beckman-Coulter). The quoted values correspond to the percentage of positive cells.

### DNA fragmentation assays

DNA fragmentation in U937 cells (2 × 10^5^) was first evaluated by agarose gel electrophoresis, as described previously [20]. Cell lysates (treated with proteinase K and RNase A) underwent electrophoresis in 1.8% agarose gels containing ethidium bromide. The gel bands were analyzed with a densitometer (Appligène-Oncor SA, Illkirch, France). DNA fragmentation was evaluated by detecting cytoplasmic histone-associated DNA fragments (mono- and oligonucleosomes) in cell lysates and supernatants from 2 × 10^4^ cells in an ELISA with anti-histone and anti-DNA fragments mAbs (Cell Death Detection ELISA^PLUS^, Roche Diagnostics, Mannheim, Germany), according to the manufacturer's instructions. Nucleosome enrichment was estimated using the streptavidin-biotin-peroxidase system and revealed by a colorimetric reaction (absorbance at 405 nm) in a microplate spectrophotometer (Bio-Rad). All experiments were performed in triplicate.

### The mitochondrial membrane potential assay

Loss of the mitochondrial membrane potential was analyzed using a mitochondrial detection kit (Biomol GmbH, Hamburg, Germany), according to the manufacturer's instructions. Following drug treatment, cells were labelled with the lipophilic fluorochrome JC-1. Depolarization of the mitochondrial membrane is characterized by a shift from red fluorescence (FL2) to green fluorescence (FL1), i.e. a reduction in the red/green fluorescence ratio. The simultaneous measurement of FL1 and FL2 was performed by flow cytometry. All experiments were performed in duplicate (at least).

### Caspase assays

Caspase-9, -8 and -3 activities in cell lysates (100 μg/assay) were assayed with specific substrates (DEVD-, IETD- and LEHD- para-nitroanilide, respectively) using caspase cellular activity assay kits (R&D Systems), according to the manufacturer's instructions. Formation of para-nitroaniline (pNA) was monitored at 405 nm. The concentrations of cleaved substrates were calculated from a titration curve established from known concentrations of pNA (nmol) (Sigma). Experiments were performed in triplicate (at least).

### Superoxide anion and ROS/RNS detection

The simultaneous production of superoxide anion (O_2_^−^) and ROS/RNS was assayed with a specific total ROS/superoxide anion detection kit (Enzo Life Sciences, Villeurbanne, France) according to the manufacturer's instructions. In brief, cells (3 × 10^5^/ml) were labelled simultaneously with the two non-fluorescent dyes that react respectively with O_2_^−^ (generating a FL2 fluorescent product), and with other types of ROS/RNS (H_2_O_2_, ONOO^−^, HO·, NO and ROO·) (generating a FL1 fluorescent product). Upon staining, the fluorescent products were quantified with a flow cytometer (Beckman-Coulter).

### The proteasome chymotrypsin-like activity assay

Proteasome chymotrypsin-like activity in cells (5 × 10^4^/assay) was measured with the specific substrate N-succinyl-Leu-Leu-Val-Tyr-aminoluciferin (Suc-LLVY- AL) by using the chymotrypsin-like cellular activity assay kit (Promega) according to the manufacturer's instructions.

### The MMP-12 activity assay

Elastase activity in whole cell lysates (cells were lysed in caspase buffer (R&D Systems)) was measured according to [92] by using Succ-Ala-Ala-Val-pNA. Free pNA was monitored at 405 nm. Recombinant proMMP-12 was diluted to a concentration of 100 mg/ml in 50 mM Tris-HCl pH 7.5 containing 150 mM NaCl, 10 mM CaCl_2_ and 0.05% (v/v) Brij^®^ (protease buffer) and activated by treatment with 1 mM p-aminophenyl mercuric acetate (APMA) for 2 h at 37°C. MMP-12 activity was assayed using the 7-methoxycoumarin-4-yl)acetyl-Pro-Leu-Ala-Gln-Ala-Val-Dpa-Arg-Ser-Ser-Ser-ArgNH_2_ peptide (a substrate for several MMPs, including MMP-12). In a typical experiment, 30–100 ng APMA-activated MMP-12 was incubated for 18 h at 37°C in 0.1 ml protease buffer containing 10 mM of the internally-quenched fluorogenic substrate, which upon cleavage at an alanine-valine bond by MMP-12 produces a fluorescent signal [(7-methoxycoumarin-4-yl)acetyl], with excitation at 314 nm and emission at 420 nm). MMP-12 activity was measured in the presence or absence of various concentrations of cysteine, NAC, BAPTA or an enzymatic O_2_^−^-generating system (100 μM xanthine and 0.1 U/ml xanthine oxidase). As controls, MMP-12-free mixtures were tested in parallel. The K_m_ and V_max_ were determined from a Lineweaver-Burk plot.

### Immunoblotting

Cells were lysed in M-PER buffer (Pierce Biotechnology, Rockford, IL, USA) supplemented with protease and phosphatase inhibitor cocktails (Sigma). Total cell extracts were separated using 7.5% or 10% SDS-PAGE, transferred to nitrocellulose membranes and blotted as described previously [93]. Immunoblotting was performed with primary antibodies diluted according to the manufacturer's instructions. Samples were then incubated with HRP-coupled secondary antibodies. Blots were visualized with an enhanced chemiluminescence kit (GE Healthcare Europe, Saclay, France) and the bands were quantified using Image J64 software.

### Statistics

Data are presented as the mean ± SD of *n* independent experiments. The statistical significance of the results was analyzed using a paired Student's *t*-test and a one-way analysis of variance (ANOVA). The threshold for statistical significance was set to *p* < 0.05 and correlations were assessed with Pearson's correlation coefficient.
